# Paraneoplastic neurological syndromes

**DOI:** 10.1186/1750-1172-2-22

**Published:** 2007-05-04

**Authors:** Jérôme Honnorat, Jean-Christophe Antoine

**Affiliations:** 1 Inserm U 842; Université Claude Bernard Lyon 1; Hospices Civils de Lyon, Lyon, France; 2 Service de Neurologie, Hopital Bellevue, CHU, Saint-Etienne, France

## Abstract

Paraneoplastic neurological syndromes (PNS) can be defined as remote effects of cancer that are not caused by the tumor and its metastasis, or by infection, ischemia or metabolic disruptions. PNS are rare, affecting less than 1/10,000 patients with cancer. Only the Lambert-Eaton myasthenic syndrome is relatively frequent, occurring in about 1% of patients with small cell lung cancer. PNS can affect any part of the central and peripheral nervous system, the neuromuscular junction, and muscle. They can be isolated or occur in association. In most patients, the neurological disorder develops before the cancer becomes clinically overt and the patient is referred to the neurologist who has the charge of identifying a neurological disorder as paraneoplastic. PNS are usually severely disabling. The most common PNS are Lambert-Eaton myasthenic syndrome (LEMS), subacute cerebellar ataxia, limbic encephalitis (LE), opsoclonus-myoclonus (OM), retinopathies (cancer-associated retinopathy (CAR) and melanoma-associated retinopathy (MAR), Stiff-Person syndrome (SPS), chronic gastrointestinal pseudoobstruction (CGP), sensory neuronopathy (SSN), encephalomyelitis (EM) and dermatomyositis. PNS are caused by autoimmune processes triggered by the cancer and directed against antigens common to both the cancer and the nervous system, designated as onconeural antigens. Due to their high specificity (> 90%), the best way to diagnose a neurological disorder as paraneoplastic is to identify one of the well-characterized anti-onconeural protein antibodies in the patient's serum. In addition, as these antibodies are associated with a restricted range of cancers, they can guide the search for the underlying tumor at a stage when it is frequently not clinically overt. This is a critical point as, to date, the best way to stabilize PNS is to treat the cancer as soon as possible. Unfortunately, about one-third of patients do not have detectable antibodies and 5% to 10% have an atypical antibody that is not well-characterized. As PNS are believed to be immune-mediated, suppression of the immune response represents another treatment approach.

## Disease name

Paraneoplastic neurological syndromes (PNS)

## Definition and diagnostic criteria

Paraneoplastic neurological syndromes (PNS) can be defined as remote effects of cancer that are not caused by the tumor and its metastasis, or by infection, ischemia or metabolic disruptions [1,2]. In most patients, the neurological disorder develops before the cancer becomes clinically overt and the patient is referred to neurologist who has the charge of identifying a neurological disorder as paraneoplastic.

In the last two decades, the discovery that some PNS are associated with antibodies directed against antigens expressed by both the tumor and the nervous system (onconeural antibodies), has suggested that these disorders are immune-mediated. Even if numerous types of paraneoplastic antibodies have been described [2-5], less than 50% of patients with PNS harbor paraneoplastic antibodies [4]. Thus, the absence of paraneoplastic antibodies cannot rule out the diagnosis of PNS.

The presence or the absence of paraneoplastic antibodies and the type of antibodies define different subtypes of PNS. Recently, an international panel of neurologists reviewed the existing criteria for diagnosis of PNS and recommended new diagnosis criteria for PNS [4]. The panel suggested two levels of evidences necessary to define a neurological syndrome as paraneoplastic: "definite" and "possible". Each level can be reached combining a set of criteria, which are based on the presence or absence of cancer, and the definitions of "classical" syndrome and "well characterized" onconeural antibody. PNS is "definite" when:

1. a classical neurological syndrome is observed (encephalomyelitis, limbic encephalitis, subacute cerebellar degeneration, sensory neuronopathy, opsoclonus-myoclonus, chronic gastrointestinal pseudoobstruction, Lambert-Eaton myasthenic syndrome or dermatomyositis) and cancer develops within five years of the diagnosis of the neurological disorder;

2. a non-classical neurological syndrome is observed and resolves or significantly improves after cancer treatment without concomitant immunotherapy, provided that the syndrome is not susceptible to spontaneous remission;

3. a non-classical neurological syndrome is observed with onconeural antibodies and cancer develops within five years of the diagnosis of the neurological disorder;

4. a neurological syndrome with well-characterized onconeural antibodies (Ab) is observed (Hu-Ab, Yo-Ab, CV2-Ab, Ri-Ab, Ma2-Ab or anphiphysin-Ab) and no cancer.

PNS is "possible" when:

1. a patient with classical neurological syndrome has no onconeural antibodies and no cancer, but has a high risk to have underlying tumor;

2. a patient presents with a neurological syndrome (classical or not) with partially characterized onconeural antibodies, and no cancer.

3. a patient has a non-classical neurological syndrome without onconeural antibodies, and cancer presents within two years of diagnosis.

## Epidemiology

PNS are rare diseases occurring in less than about 0.01% of patients with cancer [2]. Only the Lambert-Eaton myasthenic syndrome is relatively frequent, occurring in about 1% of patients with small-cell lung cancer [6].

## Clinical description

PNS may affect any level of the nervous system (central or peripheral nervous system, including the neuromuscular junction and muscle). The severity of most PNS is due to the early and non-reversible destruction of neural structures by the inflammatory process [7,8]. PNS are rapidly progressive, in many cases leaving the patients severely debilitated within weeks to months. A subacute progressive clinical course and a severe disability are highly suggestive of PNS [4]. However, slow progression, relapses or a benign course do not exclude the diagnosis.

Cerebro-spinal fluid (CSF) usually shows mild inflammatory changes and oligoclonal bands [9].

Even if some neurological syndromes suggest a paraneoplastic origin, none of them are exclusively paraneoplastic (Table 1) and non-paraneoplastic forms also exist [1]. Various antibodies have been reported in association with PNS, defining different subtypes of PNS (Table 2). However, the description of PNS antibodies may also be confusing, since a given antibody can be found in different neurological syndromes and a given syndrome can be associated with different antibodies.

**Table 1:** Main paraneoplastic neurological syndromes and associated antibodies

**Paraneoplastic neurological syndromes**	**Frequency of paraneoplastic origin**	**Main associated tumors**	**Main frequent associated paraneoplastic antibodies**
LEMS	60%	SCLC	VGCC-Ab*
Subacute cerebellar ataxia	50%	Ovary, breast SCLC Hodgkin's disease Others	Yo-Ab (PCA1-Ab**) Hu-Ab (ANNA1-Ab**) CV2-Ab (CRMP5-Ab**) Tr-Ab Ma-Ab (Ta-Ab**)
Opsomyoclonus	20%	Neuroblastoma Breast, lung	Hu-Ab (ANNA1-Ab**) Ri-Ab (ANNA2-Ab**)
Sensory neuronopathy	20%	SCLC	Hu-Ab (ANNA1-Ab**) CV2-Ab (CRMP5-Ab**)
Limbic encephalitis	20%	SCLC Testicular SCLC	Hu-Ab (ANNA1-Ab**) CV2-Ab (CRMP5-Ab**) Ma2-Ab (Ta-Ab**) Amphiphysin-Ab
Encephalomyelitis	10%	SCLC Others	Hu-Ab (ANNA1-Ab**) CV2-Ab (CRMP5-Ab**) Amphiphysin-Ab Ma2-Ab (Ta-Ab**)
Retinopathy	NA	SCLC Melanoma	Recoverin-Ab CV2-Ab (CRMP5-Ab**) Rod-bipolar-cell-Ab
Stiff-person syndrome	20%	Breast	Amphiphysin-Ab
Chronic gastrointestinal pseudoobstruction	NA	SCLC	Hu-Ab (ANNA1-Ab**) CV2-Ab (CRMP5-Ab**)

*VGCC-Ab are not really paraneoplastic antibodies because they are involved in LEMS but are not independent on the presence of a tumor

**Alternative nomenclature which can be found in the literature

**Table 2:** Main antibodies associated with Paraneoplastic neurological syndromes

**Antibodies**	**Main associated neurological syndromes**	**Cancer**
Hu-Ab (ANNA1-Ab**)	Sensory neuronopathy Encephalomyelitis Chronic gastrointestinal pseudoobstruction Cerebellar ataxia Limbic encephalitis	SCLC
Yo-Ab (PCA1-Ab**)	Subacute cerebellar ataxia	Ovary, breast, uterus
CV2-Ab (CRMP5-Ab**)	Cerebellar ataxia Sensory-motor neuropathy Uveitis, retinopathy Encephalomyelitis	SCLC, thymoma
Ri-Ab (ANNA2-Ab**)	Opsomyoclonus Cerebellar ataxia	Breast, SCLC
**Amphiphysin-Ab**	Stiff-person syndrome Sensory neuronopathy Encephalomyelitis	Breast, SCLC
**Tr-Ab**	Cerebellar ataxia	Hodgkin's disease
Ma2-Ab (Ta-Ab**)	Limbic encephalitis	Testicular
**CAR-Ab**	Retinopathy	Breast, SCLC

**Alternative nomenclature which can be found in the literature

The main neurological syndromes associated with a paraneoplastic origin and the possible role of antibodies in each case are briefly described bellow:

### Limbic encephalitis (LE)

LE is characterized by the subacute onset of confusion with marked reduction of the short-term memory. Seizures are not uncommon and may antedate by months the onset of cognitive deficit [10]. Other patients have a more insidious onset with depression or hallucinations that can lead to confusion the diagnosis with that of a psychiatric illness. Among LE patients, 50% have small-cell lung cancer (SCLC), 20% have a testicular tumor and 8% have breast cancer [11]. Hu-Ab are present in up to 50% of patients with LE and lung cancer [10,11]. A minority of patients with LE and lung cancer may harbour CV2-Ab or amphiphysin-Ab [12,13]. Ma2-Ab are present in the great majority of patients with LE and testicular cancer. Unlike patients with lung cancer, these patients usually present with diencephalic and upper brainstem symptoms that identify a characteristic syndrome in addition to limbic encephalitis [14]. Recently, VGKC-Ab have been found in some LE cases without associated cancer [15].

### Subacute cerebellar ataxia

Paraneoplastic cerebellar ataxia (PCA) is characterized by the rapid development of severe pancerebellar dysfunction due to an extensive loss of Purkinje neurons with relative preservation of other cerebellar neurons. Computed tomography (CT) and magnetic resonance imaging (MRI) studies are initially normal but demonstrate cerebellar atrophy in the later stages of the disease.

Different autoantibodies have been reported in PCA (Table 1). Yo-Ab are present in patients with PCA and ovary, breast, or other gynecological malignancies [16,17]. Tr-Ab are markers of patients with PCA and Hodgkin's disease [18,19]. Unlike Yo-Ab, Tr-Ab usually disappear after treatment of the tumor or, in a few patients, are only found in the CSF [18]. Hu-Ab are reported in 23% of patients with PCA and lung cancer [20]. PCA and SCLC is also characteristic for patients with CV2-Ab [12,21]. Zic4-Ab, Ma2-Ab and VGCC-Ab are present in nearly 40% of patients with PCA and lung cancer (usually SCLC) [22-24]. About half of these patients present a coincident Lambert-Eaton myasthenic syndrome [20].

### Opsoclonus-myoclonus (OM)

Opsoclonus is defined by the presence of spontaneous, arrhythmic and large amplitude conjugate saccades occurring in all directions of gaze, without saccadic interval. Opsoclonus is usually associated with myoclonus of the limbs and trunk, and sometimes, with encephalopathy. Paraneoplastic OM is observed in three clinical settings: 1) pediatric patients with neuroblastoma [25,26]; 2) adult female patients with Ri-Ab, whose underlying tumor is usually breast cancer [27,28], and 3) adult patients without paraneoplastic antibodies whose tumor is almost always SCLC [29,30]. There are individual case reports associated with other tumors including carcinoma of the uterus, Fallopian tube, breast, bladder, thyroid, thymus, chondrosarcoma, and Hodgkin's disease. A few patients with OM and SCLC or neuroblastoma may harbour Hu-Ab [31].

### Retinopathies

The paraneoplastic retinopathies include three well-characterized syndromes [32]. Cancer-associated retinopathy (CAR) is almost always associated with SCLC. Symptoms usually present bilaterally and reflect the simultaneous dysfunction of both cones and rods. Patients present with photosensitivity, reduced visual acuity, decreased color perception, ring scotomas, nyctalopia, and prolonged dark adaptation [33]. Ophthalmoscopic examination typically shows attenuated arterioles, and electroretinogram discloses flat or severely attenuated photic and scotopic responses. Most of the patients with CAR have recoverin-Ab, but other rare antibodies have also been described [34,35].

Melanoma-associated retinopathy (MAR) usually occurs after the diagnosis of malignant melanoma, often at the stage of metastases. Patients with MAR usually have nearly normal visual acuity and color vision but develop sudden shimmering, flickering photopsias, night blindness, and mild peripheral visual field loss [33]. Symptoms are explained by dysfunction of rods, whereas cones are not affected [36]. Typical findings in electroretinogram include a markedly reduced or absent dark-adapted b-wave, along with a slightly attenuated a-wave to scotopic stimulus. Patients with MAR usually harbor rod-bipolar-cell-Ab [37].

Uveitis, optic neuropathy or retinopathy are also observed in some patients with CV2-Ab [12,38,39].

### Chronic gastrointestinal pseudoobstruction (CGP)

Patients with CGP present with weight loss, persistent constipation and abdominal distension due to damage of the neurons of the enteric plexuses [40]. Some patients may present with dysphagia, nausea and vomiting due to esophageal dysmotility or gastroparesis, or more frequently, with severe constipation. Radiologic studies show small bowel, colonic or gastric dilatation. Esophageal manometry may reveal spasms or achalasia. The most commonly associated tumor and antibodies are SCLC and Hu-Ab or CV2-Ab, respectively. Some patients with subacute parasympathetic and sympathetic autonomic failure and prominent gastrointestinal dysfunction may also have antibodies directed against the neuronal autonomic ganglion-type of acetylcholine receptors (nAchR antibodies) [41]. In these cases, the main associated tumors are thymoma and SCLC.

### Sensory neuronopathy (SSN)

SSN is characterized by primary damage of the sensory nerve cell body of the dorsal root ganglia. A paraneoplastic origin is only one of the causes of SSN [42]. The most common low associated tumor is SCLC [43]. The main clinical complains at onset are pain and paresthesias with asymmetric distribution that involves the arms rather than the legs. Later, pain is replaced by numbness, limb ataxia, and pseudoathetotic movements of the hands. The neurologic examination shows abolition of the deep tendon reflexes and involvement of all modalities of sensation with clear predominance of the joint position. Electrophysiologic studies show marked, but not restricted, involvement of the sensory fibres [44]. Most of the patients with SSN have Hu-Ab, CV2-Ab or amphiphysin-Ab [12,13,31,42].

### Lambert-Eaton myasthenic syndrome (LEMS)

LEMS is an autoimmune disorder of the neuromuscular junction characterized by muscle weakness and autonomic dysfunction and, on electromyography, by low compound muscle action potential after nerve stimulation with decrement at low frequency stimulation (3 Hz) of more than 10% and increment after high frequency stimulation (more than 20 Hz or preferably maximal voluntary contraction) of more than 100% [45,46].

Almost 60% of patients with LEMS are paraneoplastic and SCLC is the main associated cancer, detected mostly within two years after the diagnosis of LEMS [45,46]. No serological marker for the paraneoplastic etiology exists. VGCC-Ab are present in nearly all patients with LEMS, and these antibodies do not differ between the paraneoplastic and non-paraneoplastic form. In rare cases, patients with paraneoplastic LEMS develop cerebellar degeneration [47]. In contrast, some patients with paraneoplastic cerebellar degeneration may have VGCC-Ab without clinical signs or symptoms of myasthenic muscle weakness [48].

### Stiff-Person syndrome (SPS)

SPS is a rare neurological disorder characterized by stiffness, more prominent in axial muscles, with co-contraction of agonist and antagonist muscle groups and painful spasms precipitated by sensory stimuli. Electromyography reveals the existence of continuous motor unit activity in the affected muscles at rest. Detection of GAD-Ab in almost 70% of patients suggests an autoimmune mechanism.

A paraneoplastic variant of SPS has been described in association with breast cancer in women harbouring amphiphysin-Ab [49,50]. In these patients, the onset of stiffness in upper limbs is suggestive of paraneoplastic etiology. SPS has also been reported in association with colon and lung cancer, Hodgkin's disease, and malignant thymoma.

### Encephalomyelitis (EM)

Paraneoplastic EM is characterized by involvement of different areas such as hippocampus, lower brainstem, spinal cord or dorsal root ganglia in one and the same time [4]. The clinical picture reflects the variable anatomic involvement and includes: LE, brainstem syndromes, autonomic dysfunction, myelitis, CGP or SSN. In 75% of patients, the underlying neoplasm is SCLC. SSN, LE and cerebellar ataxia are the most common clinical syndromes. The autonomic nervous system is affected in 30% of the patients (orthostatic hypotension, urinary retention, pupillary abnormalities [51], impotence and dry mouth). Most of the patients present Hu-Ab, CV2-Ab or amphiphysin-Ab [12,21,31,52].

### Dermatomyositis

Dermatomyositis is an idiopathic inflammatory myopathy with characteristic cutaneous manifestations including heliotrope rash of the periorbital skin, erythematous scaly plaques on dorsal hands with periungual telangiectasia and photosensitive poikilodermatous eruption. The myopathy is generally symmetrical and slowly progressive during a period of weeks to months affecting mainly the proximal muscles. A paraneoplastic origin is suspected in 30% of patients [53,54] and the main associated cancer are ovarian, lung, pancreatic, stomach, and colorectal cancers, and non-Hodgkin lymphoma. Many autoantibodies such as nuclear antibodies or myositis-associated antibodies have been recognized in dermatomyositis, but none are linked to tumors. The treatment of paraneoplastic dermatomyositis should be directed primarily to the tumor, but immunosuppressive treatments including corticosteroids, azathioprin and immunoglobulins are also important.

## Etiology

The discovery of onconeural antibodies has led to the widely accepted nowadays hypothesis that PNS are immune-mediated disorders. The autoimmune hypothesis in PNS is also sustained by the inflammatory CSF findings and T cell infiltration in the affected part of the nervous system revealed by pathologic examination [55]. However, the role of onconeural antibodies in neurological dysfunction is not clear.

PNS pathogenesis is currently related to an aberrant expression in the tumor of an antigen that is normally expressed only in the nervous system [56]. Even if the antigens in the tumor are identical in structure to the neural antigens, they could be recognized as foreign, leading to an immune attack directed against both the tumor and the nervous system. However, only few onconeural antibodies have been shown to play a direct role in the pathogenesis of the neurological symptoms. The role of VGCC-Ab has been demonstrated by the fact that the electrophysiologic abnormalities characteristic for LEMS can be reproduced when immunoglobulin G (IgG) from LEMS patients is given to experimental animals [57]. Recoverin-Ab are incorporated into rod photoreceptor cells and provoke cell degeneration [34,35]. In contrast, the role of the other paraneoplastic antibodies is not clear, probably because the antigens are all intra-cellular. Hu-Ab, CV2-Ab, Ri-Ab and Yo-Ab can be internalized into cell lines and can interact with their antigens *in vitro *[58-60]. However, it is not certain if these autoantibodies can cause cell lysis *in vivo *as some of the results obtained *in vitro *are controversial [61]. Moreover, immunization of experimental animals with Hu or Yo antigens induces serum antibodies but not neurological disease, and passive transfer of Yo-Ab or Hu-Ab to experimental animals does not cause disease [62]. Thus, these paraneoplastic antibodies seem to be only a marker of the autoimmunity and do not cause the disease. Recently, it has been suggested that cellular immune mechanisms play a critical role in the pathogenesis of PNS [63-65]. However, the results are not conclusive [66].

## Diagnosis and management

PNS may affect any part of the central and peripheral nervous system, the neuromuscular junction and muscle. They can be isolated or occur in association. An important point to keep in mind is that none of these disorders are specifically paraneoplastic, as each disorder can also occur in patients without cancer. The incidence of malignancy in patients with potential PNS varies depending on the disorder and ranging from 5 to 60% [67]. In almost 80% of patients, the PNS antedates the diagnosis of cancer by several months to several years. Most tumors are diagnosed within 4–6 months. It is accepted that the risk of cancer development decreases significantly two years after diagnosing PNS and becomes very low after four years. All these data explain why it is often difficult to recognize a given neurological disorder as paraneoplastic.

It has been shown that several PNS are associated with onconeural antibodies. Unfortunately, almost 50% of patients with true PNS do not have any of the well-characterized onconeural antibodies [4]. In these patients, early diagnosis of the tumor is frequently difficult, resulting in a significant delay in tumor treatment [31]. To date, the best way of stabilizing a PNS is probably to treat the tumor as soon as possible. An early diagnosis of a neurological syndrome as PNS is thus crucial for the management of patients. Detection of an onconeural antibody in a patient suspected to have a PNS is, at present, the most valuable diagnostic test [4].

### Identification of the tumor

For patient with a neurological disorder that is highly suspected to be paraneoplastic but for whom no cancer has yet been identified, detection of the tumor is essential but may be difficult since the tumors initially may be histologically small and localized [68]. If paraneoplastic antibodies are present, they direct the tumor search to specific organs. If a SCLC is suspected, the tumor is generally detected by chest CT scan [69,70]. If a gynecological tumor is suspected, careful breast and pelvic examination, mammography and pelvic CT scan are recommended. If no malignancy is revealed during initial workup, surgical exploration and removal of pelvic organs may be warranted, particularly in postmenopausal women [16]. The use of whole-body positron emission tomography with fluorodeoxyglucose (FDG-PET) should be reserved for patients with paraneoplastic antibodies for whom conventional imaging has failed to identify a tumor or when lesions are difficult to biopsy [68,71]. In patients without paraneoplastic antibodies, the sensitivity and specificity of FDG-PET are less significant [72]. If a tumor which does not fit the known tumor pattern associated with PNS is identified, it should be checked for atypical expression of the relevant antigen [31] and the possibility of a second malignant disorder must be considered [73].

### Treatment

The different subtypes of PNS are defined by the presence or the absence of paraneoplastic antibodies and the type of antibodies. Management and treatment should be tailored to each subtype. For example, patients with SCLC and PCA have different course of the neurological disorder, response to treatment, tumor prognosis and cause of death, according to the presence or the absence of Hu-Ab [20]. Patients with Ma2-Ab generally develop limbic and brainstem encephalitis with testis tumors, but patients with Ma2-Ab and additional antibodies directed against other Ma proteins develop additional cerebellar symptoms and have tumors other than testis neoplasms [23]. The mean survival time for patients with SCLC and CV2-Ab is 2.5 times longer than for patients with the same cancer, similar neurological symptoms and Hu-Ab [74]. All these data suggest that the type of the immunological stimulation against the tumor can determine a specific course of the neurological disorder and give information of whether the immunological response against the tumor is more or less effective.

However, PNS rarely improves with immunomodulatory treatment as in the majority of cases the neurological symptoms are due to neuronal damage. The early and non-reversible destruction of neural structures by the inflammatory process accounts for both the severity of most PNS and for the usual ineffectiveness of immunomodulatory treatments [7,8]. The best chance of at least stabilizing the syndrome is to induce a complete response of the tumor [7]. Although immunotherapy is rarely effective, the use of intravenous immunoglobulins, steroids or plasmapheresis is indicated because a few patients have been reported to improve with these treatments [7,8,75]. Patients with Tr-Ab and Ma2-Ab and testicular cancer are more likely to improve than those with other antibodies [14,18].

## Unresolved questions

To date, no studies have conclusively proved that paraneoplastic antibodies are pathogenic; nevertheless, the paraneoplastic antibodies are useful diagnostic markers that can be used also to classify the different subtypes of PNS. Some studies suggest that PNS with Yo-Ab, Ma-Ab or Hu-Ab are probably autoimmune diseases directed against Yo, Ma and Hu antigens respectively, and may be due to T cell mediated destruction of neurons. However, further studies are still required in order to characterize the mechanisms leading to neuronal death in PNS. Thus, the hypothesis that PNS are immune-mediated remains to be proved.

## Conclusion

Finally, elucidation of the pathogenesis of PNS may have important implications for understanding the neuronal degeneration as well as tumor and brain immunology. As PNS are rare, the diagnosis is difficult and frequently the patient is misdiagnosed. Neurological symptoms usually develop before the diagnosis of tumor that is in early and limited stage and can be better treated by the current therapies.
